# "Squint" and Spectacles

**Published:** 1889-08-10

**Authors:** 


### "SQUINT" AND SPECTACLES.

A recent writer on ophthalmic surgery calls attention to the fact that many cases of "squint" in children, which, if left to themselves, become so pronounced that only a surgical operation can be of service to them, would be easily cured by the use of proper spectacles if seen by a competent specialist in the earlier stages of the affection. The present generation, he says, has witnessed many improvements in the operation for squint. The objects to be aimed at by operations have become well understood. But it is stated that Board schools and other educational establishments are still busily engaged in manufacturing fresh cases, though, thanks to improved spectacles, there are now fewer squints requiring operation than formerly. Age is hardly a bar to the wearing of spectacles, quite young children soon becoming accustomed to their use. It is possible that enthusiastic specialists may sometimes carry their principles too far. The sight of so many boys and girls in streets, and schools, and offices with "spectacles on nose" is not encouraging. Still, if many of the youthful patients are merely undergoing a temporary treatment for squint there is less reason for regret. Undoubtedly, it is better for a child to wear spectacles for a few years, and thus to be cured, than to have to run the risk of tendon section in later life.

